# Independent Positioning and Action of *Escherichia coli* Replisomes in Live Cells

**DOI:** 10.1016/j.cell.2008.01.044

**Published:** 2008-04-04

**Authors:** Rodrigo Reyes-Lamothe, Christophe Possoz, Olessia Danilova, David J. Sherratt

**Affiliations:** 1 Department of Biochemistry, University of Oxford, Oxford OX1 3 QU, UK

**Keywords:** DNA, CELLCYCLE, MICROBIO

## Abstract

A prevalent view of DNA replication has been that it is carried out in fixed “replication factories.” By tracking the progression of sister replication forks with respect to genetic loci in live *Escherichia coli*, we show that at initiation replisomes assemble at replication origins irrespective of where the origins are positioned within the cell. Sister replisomes separate and move to opposite cell halves shortly after initiation, migrating outwards as replication proceeds and both returning to midcell as replication termination approaches. DNA polymerase is maintained at stalled replication forks, and over short intervals of time replisomes are more dynamic than genetic loci. The data are inconsistent with models in which replisomes associated with sister forks act within a fixed replication factory. We conclude that independent replication forks follow the path of the compacted chromosomal DNA, with no structure other than DNA anchoring the replisome to any particular cellular region.

## Introduction

The ubiquity of DNA as the genetic material generates a spatial dilemma: compact and organize a molecule at least a thousand times longer than the cell or nucleus that contains it and yet retain the ability to use it. Bacteria solve the problem of DNA compaction-organization by using a combination of supercoiling, chromosome-associated proteins, counterions, and excluded volume effects (Woldringh and Nanninga, 2006; Zimmerman, 2006). The first level of organization of bacterial chromosomal DNA is into ∼10 kb topologically independent supercoiled domains (Deng et al., 2005; Postow et al., 2004). Genetic loci occupy predictable cellular positions that change over time (Nielsen et al., 2006b; Viollier et al., 2004; Wang et al., 2005, 2006). The 4.6 Mbp *E. coli* circular bacterial chromosome is organized about a transverse axis, with nonreplicating cells having the *ori* region close to midcell, the left and right arms of the chromosome positioned on either side, and the *ter* region crossing between the outer nucleoid edges.

Since bacterial chromosome segregation can occur sequentially and progressively as DNA replication proceeds, with the time it takes a pair of sister forks to replicate the chromosome being much longer than the generation time, it is important to determine how DNA replication is organized within the compacted nucleoid, whose DNA is being continually remodeled as replication-segregation proceeds.

The *E. coli* chromosome is replicated once per cell division, from a unique replication origin, *oriC*. Replication proceeds bidirectionally until the two replication forks meet, normally in a broad terminus (*ter*) region. Replication of DNA is carried by a group of at least 13 proteins that form a functional unit, the replisome (O'Donnell, 2006). A replisome associates with each fork, where it polymerizes both the leading and lagging strands.

A body of experimental evidence has been used to support the view that in bacteria the two sister replisomes derived from a given initiation event are associated into a replication factory (Adachi et al., 2005; den Blaauwen et al., 2006; Lemon and Grossman, 1998, 2000; Molina and Skarstad, 2004). Nevertheless, there is no functional dependency between two elongating forks (Breier et al., 2005; Possoz et al., 2006), and transient or stable separation of sister forks has been reported (Bates and Kleckner, 2005; Berkmen and Grossman, 2006; Hiraga et al., 2000; Kongsuwan et al., 2002; Migocki et al., 2004).

By marking different *E. coli* replisome components, the fate and dynamics of replisomes, relative to genetic loci, has been tracked in live cells from replication initiation to termination. We show that the replisome assembles at *oriC* at the time of initiation, irrespective of *oriC* position in the cell. Furthermore, the two forks separate into different cell halves ∼5 min after initiation and come together at midcell before disassembly at replication termination. The results support a model proposed to explain the observed < left-*ori*-right-left-*ori*-right > organization of *E. coli* sister chromosomes (Wang et al., 2006) and are inconsistent with models of fixed replication factories that contain both replisome-associated forks. Rather they suggest that the replication machinery at each sister fork acts independently as it tracks along DNA.

## Results

### Fluorescent Replisome Foci Mark Replication Forks

Fluorescent C-terminal fusion-protein derivatives of *E. coli* replisome components, expressed from their endogenous chromosomal promoters, were constructed and their phenotypes assessed by growth, flow cytometry, and microscopy. Fusions containing components of the PolIII holoenzyme, the clamp loader, and Ssb (single-strand binding protein) were chosen for more detailed study because they showed growth and chromosome processing parameters indistinguishable from wild-type (Figure S1 available online).

**Figure 1:**
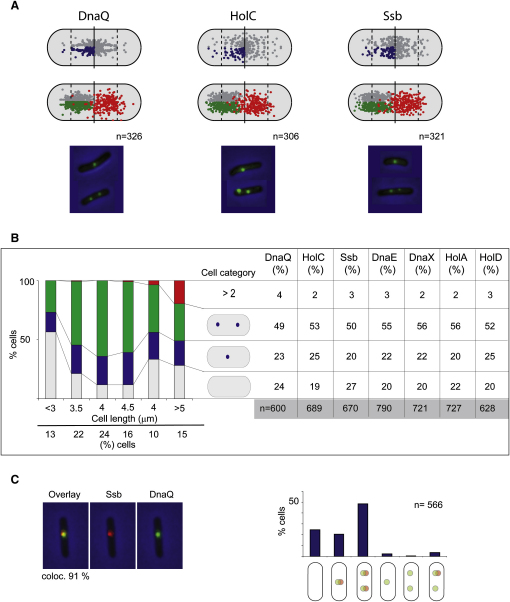
Replisome Localization (A) The cellular distribution of DnaQ, HolC, and Ssb foci. The distribution of cellular focus positions is shown for cells with one focus (blue) or two foci (green and red). The distance to the closest pole and side was determined for one focus cell. For cells with two foci, the most polar focus was always considered as focus 1 (green). The blue and green focus quartile positions are shown expanded to the whole cell and half cell, respectively, in gray. When two foci are present, their relative positions are conserved. (B) Number of replisome foci as a function of cell length. A cell population was divided into groups based on cell length and the proportion of cells with 0, 1, 2, and >2 DnaQ foci is presented for each group. The % cells with/without foci for the assayed replisome proteins is shown. HolA, C, and D are clamp loader components; DnaE is the replicative polymerase, PolII; DnaX is the replisome organizer/clamp loader. (C) Colocalization of Ssb and DnaQ. For a subpopulation of cells containing Ssb-CFP and DnaQ-YPet, the proportions of cells in the indicated categories are shown. The probability of a Ssb focus colocalizing with DnaQ was 91%.

Initial snapshot analysis of cells growing with a ∼100 min generation time at 37°C documented the number and positions of seven replisome markers (Figures 1A and 1B). Previous work has shown that under these conditions, DNA replication initiates soon after birth in most cells, with a proportion of cells initiating replication just before cell division (Wang et al., 2005).

Cell populations lacking a replisome focus (19%–27%; depending on the replisome component) were enriched for shorter cells (<3.5 μm long) that had presumably not yet initiated replication and longer cells (>4.5 μm long), which were expected to have completed replication (G2). Cells containing two replisome foci (49%–56%) were predominant in the cell population of intermediate length, precisely those expected to be undergoing DNA replication (S phase). Although cell length does not give a precise measure of cell age, the correlation is generally good when snapshot and timelapse analysis is compared (later).

Single focus cells (20%–25%) had the focus positioned in the midcell region (>85% in the midcell third), while those with two foci generally had the foci in different cell halves, with the mean position close to the nucleoid quarter. By labeling Ssb with CFP, it was demonstrated that the positions of Ssb foci were coincident with those of DnaQ (the 3′–5′ exonuclease subunit of PolIII), HolC, DnaE, and HolD (Figure 1C and data not shown; >90% colocalization). Therefore, all replisome components tested occupy the same cellular position at a given time, giving us confidence in both reagents and assays. The simplest interpretation of these data is that a single focus represents two forks close together or individual replication forks that move apart to separate cell halves after replication initiation.

### Replisome Foci Appear at the Time of Replication Initiation

**Figure 2:**
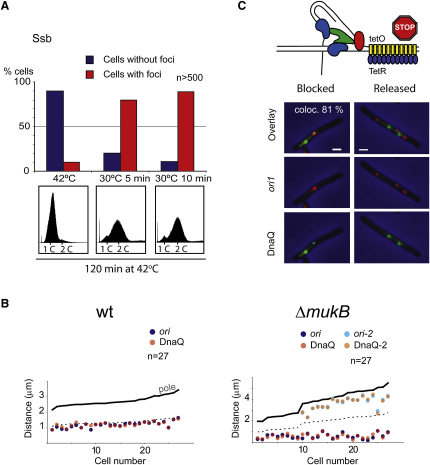
Replisome Foci Appear at *oriC* on Replication Initiation (A) Initiation and focus appearance. The proportion of cells with/without Ssb foci is shown for cells carrying the *dnaC2* allele when incubated at 42°C for one generation and 5 and 10 min after transfer to 30°C. For flow cytometry analysis, cultures were incubated for 120 min at 42°C after the indicated treatment to allow completion of DNA replication. (B) Timelapse (5 min intervals) analysis of wild-type (left) and Δ*mukB* cells (right). At the time of appearance of DnaQ foci, the correlation between DnaQ position at replication initiation and *ori1* position was recorded. In Δ*mukB* cells, *ori*1 is aberrantly positioned close to the poles, immediately prior to initiation. Δ*mukB* cells were grown at 22°C and wild-type cells at 37°C. Foci are shown for cells sorted by length. Note that Δ*mukB* cells are often delayed in cell division and reinitiate replication prior to division. (C) Colocalization of replisome and stalled fork. Cells carrying a *tetO ori1* array were induced to overexpress TetR-CFP to block replication at *ori1*. Localization of DnaQ was analyzed in long cells with one *ori* focus to ensure that they were blocked. The probability of colocalization of DnaQ and an *ori1* focus was 81%. The presence of >2 *ori* foci in the cell shown is a consequence of new initiations occurring during the period of the block. Bar, 1 μm.

To assess whether replisome components assemble prior to replication initiation, strains carrying *dnaA46* or *dnaC2* alleles, which have a temperature-sensitive initiation protein, DnaA, or a temperature-sensitive replisome loader protein, DnaC, respectively, were used to visualize replisome assembly. These strains can complete replication but cannot reinitiate replication when grown at the restrictive temperature (Withers and Bernander, 1998). After growth at 42°C for 100 min, >90% of cells lacked Ssb or DnaQ foci (Figures 2A and S2A) and had completed replication, as judged by flow cytometry. In the DnaC^ts^ strain, the small minority of cells with foci at restrictive temperature are likely to be cells in which the replisome remains at a stalled or broken fork (Maisnier-Patin et al., 2001).

After a 5 min shift to 30°C, most DnaC^ts^ cells had reinitiated replication when assessed by flow cytometry and 82% had gained an Ssb focus. After 10 min at 30°C the proportion of cells with Ssb foci increased to ∼90%. Similar results were obtained when DnaQ was analyzed (Figure S2A). With the DnaA^ts^ strain, the frequency of reinitiated cells was somewhat lower, but Ssb and DnaQ foci appeared only on reinitiation. We conclude that replisome assembly requires functional DnaA and DnaC and that the appearance of DnaQ or Ssb foci reports replication initiation. In the absence of functional DnaA and DnaC, we saw no evidence for a pre-replicative complex containing the replicative polymerase (DnaQ) or Ssb. Furthermore, DnaQ foci were absent in Muk^−^ cells lacking DNA (data not shown).

### The Replisome Assembles on *oriC* at Initiation

Timelapse tracking in living cells of DnaQ or Ssb (Figures 2B and S2B), with respect to the *ori1* locus (16 kb anticlockwise of *oriC*), showed that at the time of appearance of a new replisome, its position correlates strongly with that of *ori1*, which marks *oriC*. Furthermore, snapshots of cells containing single *ori1* and replisome foci show that they exhibit a strong positional correlation, which disappears once either marker focus has duplicated (data not shown).

These results, along with our earlier data, indicate that the replisome assembles at *oriC* at the time of initiation, rather than *oriC* moving to an assembled pre-replicative complex (Bates and Kleckner, 2005). In order to test this more rigorously, we exploited a MukBEF^−^ strain in which *ori1* is located at the old pole rather than at midcell at birth, in cells grown at 22°C (Danilova et al., 2007). Simultaneous timelapse visualization of *ori1* and DnaQ showed that the replisome first appears at or close to *oriC* irrespective of whether its position is normal at midcell or abnormal close to the old pole (Figure 2B). The polar position of *ori1* in Muk^−^ cells is established before the replisome appears at *ori1* at initiation, since 85% of *ori1* foci move <0.2 μm in the 5 min prior to replisome appearance. Taken together, these results demonstrate conclusively that the replisome assembles at *oriC*, irrespective of its position, rather than *oriC* moving to the replication machinery.

### Replisomes Remain at Replication Forks Stalled by Tightly Bound Repressor

We have previously shown that LacI or TetR repressors tightly bound to arrays of their cognate sites leads to efficient site-specific, yet rapidly reversible, replication fork stalling, with Ssb remaining associated with stalled forks (Possoz et al., 2006). To test whether other components of the replisome mark the blocked fork, we tested DnaQ localization in cells with a blocked *ori1* array.

Replication blockage by tight TetR-CFP binding to *ori1* was induced by loading the cells with repressor expressed from a multicopy plasmid and then removing anhydrotetracycline (AT), whose presence relieves tight binding. After 100 min incubation at 37°C, DnaQ and *ori1* were visualized in blocked cells and in cells in which the block was released by AT addition for 20 min (Figure 2C). Blockage and the subsequent release were efficient: 59% of blocked cells contained a single *ori1* focus, as compared to 32% in the nonblocked control. After release of the block the proportion of single *ori1* focus cells had reduced to 13%. The great majority of blocked *ori1* foci colocalized with DnaQ (81%), confirming that the replisome foci mark the location of the blocked replication forks.

### Replisome Positioning over Time

**Figure 3:**
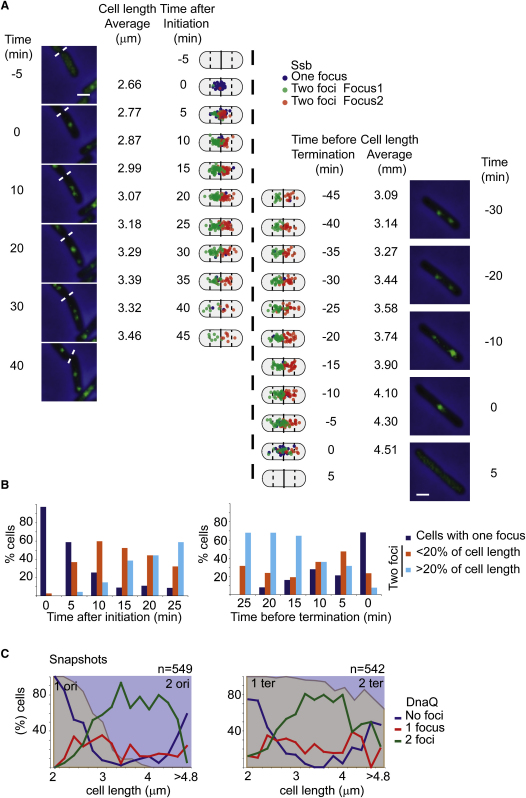
Replisomes and the Cell Cycle (A) The localization of Ssb was followed in 50 cells every 5 min for 60 min. Timelapse series were made according to the time after the appearance or the time before the disappearance of Ssb foci and are shown as two overlapping series. Cells having one focus are represented as blue dots, while cells with two foci are shown in green (first) and red (second). A set of images 10 min apart is shown for two representative cells. Bar, 1 μm. (B) Statistics of appearance, disappearance, and movement apart of Ssb foci at replication initiation and termination. (C) Snapshot analysis of DnaQ with respect to *ori1* (left panel) and *ter3* (right panel). A steady-state cell population was separated into classes according to cell length. The proportion of cells with one or two *ori1* foci (gray or blue areas, respectively) and with zero, one, or twp DnaQ foci (blue, red, or green lines, respectively) is represented for each of the classes (left panel). A comparable analysis was done with DnaQ and *ter3* (right panel).

Analysis of Ssb in fifty timelapse series (5 min intervals) gave a pattern consistent with that inferred from snapshots. Images of two overlapping series and the patterns for all 50 series are shown, as are individual lineage traces (Figures 3A and S3). Foci appear either shortly after birth or shortly before birth in a mother cell and are present for 65 min on average. Assuming the times of appearance and disappearance of foci mark replication initiation and termination, respectively, then this value for S phase, determined for timelapse cells growing on slides, is slightly longer than our previous estimate of ∼55 min for cells growing in the same liquid medium (Wang et al., 2005).

The appearance of a single Ssb focus occured close to midcell (0 min after initiation). By 5 min, this single focus was replaced by two separated foci in 59% of the cells, while 10 min after initiation, 76% of cells had two Ssb foci (Figure 3B). Therefore, the two forks and their associated replisomes separate early during the elongation phase of replication. Five minutes of replication corresponds to ∼180 kb DNA replicated for each fork, ample time to give the observed spatial separation of two independent forks. After separation, the two foci almost always occupy different cell halves, with both replisomes being mobile. Snapshot analysis of *ori1* and *ter3* loci, with respect to the replisome marker DnaQ, confirmed this overall picture (Figure 3C). For example, cells having one *ori1* focus and no DnaQ focus (19%) have presumably not established an active replisome at the origin because they are in G1. Although the majority of mid-sized cells with two *ori1* foci have two DnaQ foci (∼80%), there is a minority of cells (∼20%) with two *ori1* foci and one replisome focus; this is likely a consequence of two forks transiently moving close together and appearing as a single focus before splitting again into two foci, as observed by timelapse (Figure 3C).

The timelapse analysis shows that toward the end of replication, Ssb foci move closer to the cell center, with 62% of the cells at the last time point before focus disappearance having one focus located in the middle third of the cell, as compared to 18% 5 min earlier (Figure 3B). This pattern was also evident in the snapshot analysis, with longer cells having zero or one DnaQ foci, the increase in zero replisome cells being mirrored by an increase in cells with duplicated *ter3* foci (Figure 3C), because replication had terminated and replisomes had disassembled prior to *ter3* focus duplication.

### Sister Chromosome Cohesion

**Figure 4:**
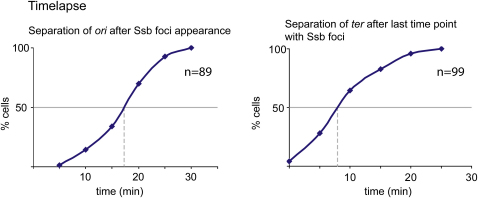
Sister Cohesion The time of separation of sister *ori1* foci (left panel) or *ter3* foci (right panel), with respect to time of Ssb appearance or disappearance, respectively, is plotted. In the right panel *ter3* separation is related to the last time at which Ssb is present.

Timelapse analysis (5 min intervals) showed that 50% of cells had separated *ori1* foci 17 min after the appearance of the replisome focus (Figure 4, left), a value consistent with the snapshot analysis, if one relates cell length to cell age (Figure 3C). Since the newly separated sister replisomes have moved away from *ori1* by 5 min after replisome appearance, we infer that *ori1*, which replicates ∼0.5 min after initiation, will have been replicated within 5 min of replisome appearance. Therefore replication initiation should occur 0–4.5 min after replisome appearance, consistent with flow cytometry, which showed that most cells had initiated replication within 5 min of replisome appearance (Figure 2A). Therefore, the time between *ori1* replication and its visible separation into two foci is in the range 11.5–16.5 min.

The analysis also showed that 50% of *ter3* loci had separated by 8 min after the last time at which a replisome was present (Figure 4, right). Therefore the replisome disappears 3 min on average before *ter3* sister focus separation. Assuming that the disappearance of replisome foci marks replication completion and that termination occurs most often in the 300 kb region flanked by the replication termination sites, *terC* and *terA*, completion of replication will normally be within 5 min of replication of *ter3*, if both forks are approaching the *ter* region simultaneously. Therefore, there will be a lag of 3–8 min after replication of the *ter3* locus and its visible segregation, assumimg no lag in completion of replication because of a retarded fork. Any replication lag in the fork that does not replicate *ter3* would extend this apparent cohesion time. By comparison, we estimate below a cohesion period of ∼10 min for L3 and R3. We conclude that the cohesion periods for *ori1*, *ter3*, L3, and R3, measured more directly than previously, are broadly similar and represent a small fraction of S phase and the generation time.

### Replisome Position Is Highly Dynamic

**Figure 5:**
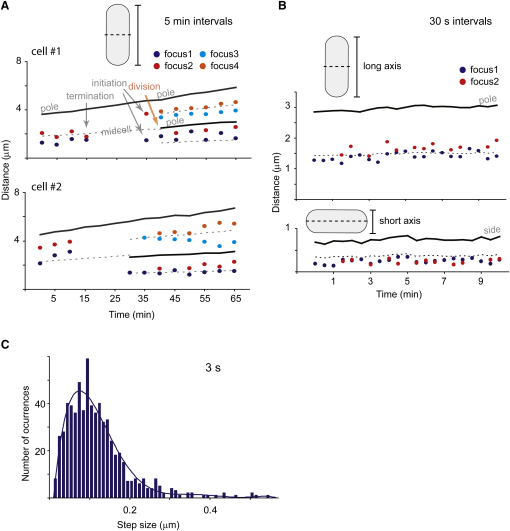
Replisome Position Is Highly Dynamic (A) Movement of Ssb foci in 5 min intervals. Timelapse series for two cells show the position of Ssb foci on the long axis of the cell through time. In cells with one focus this is represented by a blue dot, and the second, third, and fourth foci are represented with red, light blue, and orange, respectively. (B) Movement of Ssb foci in 30 s intervals. The position of Ssb foci with respect to the cell long and short axes is shown. (C) Distribution of step sizes (long axis) in 3 s timelapse.

Because the initial timelapse tracking of Ssb showed that replisome positioning is dynamic (Figures 3 and S3), a more extensive 3 s, 30 s, and 5 min analysis was undertaken, with focus position with respect to both the transverse and longitudinal cell axes being tracked in the 30 s analysis (Figure 5). The positions of foci changed continually over time, with movement along the long axis greater than that along the short axis. In the short axis analysis shown, the focus positioning on one side of the long axis was not a consistent pattern. During S phase, two foci are present most of the time, with some oscillation between one and two foci. For example, in the 21 consecutive 30 s images in Figure 5B (long axis), there are 11 fusion-splitting events, with two foci present at 12 times. By comparison, we infer from the data in Figure 2B and Figure 4 that two replisome foci are present ∼80% of the time in S phase in a cell population.

The disappearance of replisome foci at termination and the reappearance at initiation are seen for both cells in Figure 5A, cell #1 reinitiating synchronously prior to septation, while for cell #2, the two daughter cells initiate asynchronously after their birth. Such asynchronous initiation in newly born daughter cells is common under timelapse growth conditions on a slide, whereas reinitiations prior to septation are always synchronous within the time resolution of the experiments.

There was considerable replisome movement between consecutive images in the 3 s analysis, with sister replisome foci being similarly mobile. For the cell shown in Figure 5B, the average movement was 195 nm/30 s along the long cell axis. Some steps were of more than 500 nm/3 s (Figure 5D), giving an accumulated distance of more than the length of the cell during the 10 min experiment. The mean step size along the long axis was ∼100 nm/3 s.

### Replisome and Chromosome Movement Are Linked

The dynamic nature of the position of DNA replication in the cell and the separation of the two forks for much of the cell cycle argue against the idea of a structure restricting the replication machinery to a particular place in the cell and raise the possibility that each replisome independently tracks on DNA. Segments of chromosomal DNA are highly dynamic in vivo (Fiebig et al., 2006), with the expectation that the movement is larger when a locus is being segregated after replication, when the cell has to reorganize the remaining part of the parental molecule and to accommodate the newly synthesized molecules. If a replisome is indeed tracking DNA, its position will be determined both by the movement of the replisome fork on the chromosome and by the movement of the DNA segments within the nucleoid that the replisome fork is associated with at any instant in time.

To test the relationships between movement of chromosomal loci and the replication forks, we analyzed replisome positioning with respect to loci L3 (2268 kb) and R3 (852 kb; Figure S4), normally replicated at about the same time by separate forks, ∼33% of S phase before termination. If the replisomes are tracking along DNA, replisome movement will not only reflect the movement of the DNA with which it is associated but also exhibit additional movement associated with its passage along the chromosome. In contrast, if DNA is passing through a replisome, the movement of the replisome is likely to be less than that of the DNA it is associated with.

**Figure 6:**
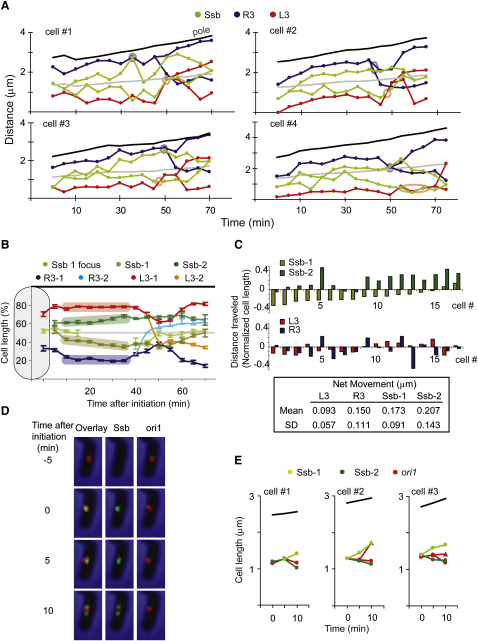
Relative Movement of Replisome and Genetic Loci (A) The position of Ssb relative to loci L3 and R3 was followed at intervals of 5 min in 21 timelapse series (also see Figure S4). (B) Mean focus positions are graphed with standard errors of the mean (SEMs) indicated by bars. The shaded areas emphasize the position of the loci relative to the replisome in the period before L3-R3 replication. (C) Net movements of Ssb with respect to L3-R3 from first appearance of the replisome to 35 min later. SD = standard deviation. (D) Relative movement of Ssb with respect to *ori1* and initiation. (E) Relative movement of Ssb and *ori1* in three cells.

Twenty-one cells in which appearance of a replisome at replication initiation to their disappearance at termination could be followed in timelapse analysis (5 min intervals) were analyzed (Figures 6A and S4). The integrated patterns of behavior are shown in Figures 6B and 6C. L3 and R3 reside close to opposite outer nucleoid edges for most of the time from initiation to replication, as described previously (Wang et al., 2006). The single replisome focus, close to midcell, splits into two soon after initiation, with the sister replisomes moving to separate cell halves and with an average progression outwards of each replisome as replication proceeds. Therefore, the overall trend of replisome movement is consistent with replisome tracking along the DNA since genetic loci are placed progressively outwards as one moves from *ori* to *ter* (Wang et al., 2006). The precise net distance traveled by replisomes varied between replisomes and from cell to cell, with a mean relative movement apart for sisters of 0.38 of a normalized cell length in the first 35 min of S phase (Figure 6C). In 13/17 cells, there was significant net relative movement of each sister replisome of a pair (for example, cells #1 and #2 in Figure 6A), whereas in 4/17 cells most net movement was the result of movement of either the left or right replisome (for example, cell #3, or the top left cell in Figure S4). Even in these latter cases, the net nonmobile replisome showed significant movements in individual 5 min steps (Figures 6A and S4). Given the observation that replisomes can move >30 nm/s, these differences between cells are not surprising. Examination of all Ssb lineages (Figures 5A, 5B, 6A, S3, and S4) provides compelling evidence that sister replisomes are similarly mobile with respect to midcell and their starting position.

Importantly, mean L3 and R3 movement in the 35 min period after initiation was less than the replisome, although abrupt large movements of L3 or particularly R3 frequently occurred close to replication initiation and locus replication segregation (Figures 6A, 6C, and S4); in part this may be due to nucleoid remodeling as replication proceeds.

Separation of L3 and R3 foci occurred on average at 53 min (R3) and 55 min (L3) after initial replisome appearance, ∼9 min and ∼11 min after we expect the loci to be replicated. Replisome and genetic locus were always close to coincident during this period. In 17/21 cases, segregation gave the < L-R-L-R > pattern observed previously (Wang et al., 2006). At about the time of L3-R3 focus separation, the locus/sister loci moved away from the outer edge of the nucleoid and toward midcell. We believe this is a consequence of the loci being close to the *ter* region, which is replicated last, rather than a necessary requirement for their replication segregation. We note that in ∼20% of the cells, at least one of the genetic loci retained its polar position both at the time of closest proximity to the Ssb foci and at the time of locus separation. Therefore, replication is not constrained to the middle third of the cell, and the inwards movement of genetic loci prior to their separation appears not to be linked to their replication.

The behavior of replisomes with respect to *ori1* after replication initiation was also analyzed in 5 min timelapse experiments (Figures 6D and 6E). In the three analyses shown, during a 5 min interval, a single Ssb focus moves away from its position coincident with *ori1* to sister positions >250 nm apart and distinct from that of *ori1*; both replisomes move relative to the starting position. Note that the separation of sister *ori1* foci can be >400 nm in a 5 min interval (cell #2), this segregation-associated movement being much greater than for a nonsegregating locus (compare Figures 6C and 6E). The separation of sister replisomes was prevented in the presence of the DNA synthesis inhibitor HU, and a single replisome focus colocalized with *ori1* for at least 45 min after replication inhibition (Movie S1). These data add support to the view that replisomes track along DNA during replication, when they are more mobile than genetic loci. In contrast, during replication inhibition, colocalized replisome and *ori1* move together.

**Figure 7:**
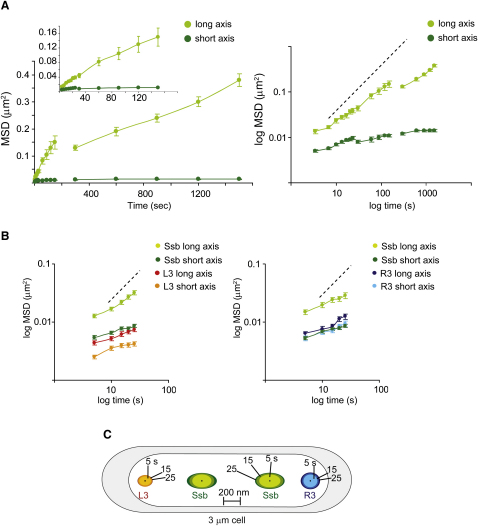
The Replisome Is More Mobile than L3-R3 Genetic Loci (A) Dynamics of Ssb plotted as MSD against time. Left panel is linear plot and right panel is log plots. The broken line shows the curve expected for free diffusion (slope 1). Error bars represent SEM. (B) Relative movement of Ssb, L3, and R3 in long and short cell axes. Log plots only are shown. Linear plot data were used to compute apparent diffusion coefficients, D*app*. Error bars represent SEM. (C) Cartoon of a cell is shown in which ovoids of different colors represent the average distance traveled by the loci L3, R3, or Ssb in 5 s, 10 s, 15 s, and 25 s (inner to outer layers). Half of the length or width of the ovoid represents the average distance traveled in the long axis or short axis in the indicated times.

To quantitate replisome dynamics further and relate them to dynamics of L3-R3 genetic loci, focus movement in each time step was measured and then the accumulative movement over time plotted as MSD (mean-square displacement) versus time (Figure 7), using the data from 3 s, 30 s, and 5 min timelapses (Figures 5 and 6). By analysis of linear-linear and log-log plots, one can distinguish diffusive, restrained, and directed motions over time and calculate an apparent diffusion coefficient for the diffusive component of any motion (Elmore et al., 2005; Fiebig et al., 2006).

The dynamics of Ssb in the three data sets (Figures 5 and 6) were similar. In each case, the log MSD (long axis) versus log time plots show evidence of restrained behavior, as expected for a DNA-associated replisome; segments of polymeric DNA are restrained in their diffusion (Figure 7). The slopes of the log-log curves were 0.7, 0.66, and 0.58 for replisome movement in the long axis in the three experiments, less than a slope of 1 expected for free diffusion (Fiebig et al., 2006). This restraint is also evident in the MSD versus time plots (Figures 7A and 7B), where the initial slope is higher than the final slope. The initial linear slopes give an MSD/t of ∼(10^3^) nm^2^/s for the replisome in all three experiments and an apparent one-dimensional diffusion coefficient (D_app_) of ∼(5 × 10^2^) nm^2^/s for movement along the long axis.

In contrast to Ssb, the dynamic behavior of L3 and R3 along the long axis, prior to their replication, is more restrained. This is evident from the log MSD versus log time plots, where the slope is ∼0.25 for L3 and ∼0.35 for R3, and by comparison of the initial slopes of the MSD versus time plots; L3 has a slope about one-tenth that of the replisome in the long axis, an MSD/t of ∼(10^2^) nm^2^/s, and a D_app_ ∼(5 × 10^1^) nm^2^/s. The mobility of R3 was somewhat higher than L3 in the long axis but still many times lower than that of the replisome; MSD/t was ∼(1.4 × 10^2^) nm^2^/s and a D_app_ of ∼(7 × 10^1^) nm^2^/s (Figure 7C). These values for L3 and R3 are similar to those determined for origins in *E. coli* and *Vibrio cholera* and for genetic loci in yeast (Elmore et al., 2005; Fiebig et al., 2006; Marshall et al., 1997).

Movement of both the replisome and L3-R3 in the short axis of the cell is more restrained than that in the long axis. L3 shows a D_app_ 2-fold lower than that in the long axis, while that of the replisome is ∼5-fold lower than that in the long axis. The differences in the dynamics of L3-R3 and the replisome are shown schematically in the cell cartoon (Figure 7C) and strengthen the view that the replisome tracks along DNA and that the replication fork in DNA is the only determinant of replisome position.

Finally, we used the 3 s and 5 s timelapse data in Figure 7 to compute the correlation in individual step sizes for a given replisome and its sister for each time point. This analysis showed that in >50% of 3 s or 5 s steps, the long axis movement of a given replisome was in a 2-fold range of that of its sister in the same interval, thereby confirming that sister replisomes exhibit similar dynamics.

## Discussion

### DNA Replication and the Cell Cycle

The work reported here has uncovered novel features of the organization of *E. coli* DNA replication and its relation to the cell cycle. Analysis of living cells that have only a single pair of replication forks, with a complete round of replication occurring in the absence of division, has shown that different replisome reporters colocalize and mark both active and stalled replication forks. By tracking the position in space and over time of replisome components and relating them to genetic loci, we have shown that in the presence of initiation proteins, DnaA and DnaC, the replisome assembles at *oriC* immediately prior to initiation, independently of the position that *oriC* occupies in the cell. By 5 min after initiation, the two sister replication forks have separated spatially into different cell halves, where they act independently during DNA synthesis. Separation of sister replisomes after initiation precedes separation of the newly replicated origin regions by ∼9 min. Toward the end of DNA replication the two replisomes come together at midcell, before dissociating at replication termination. The position of the forks is highly dynamic and appears unconstrained by cellular structures other than DNA. The data are not consistent with replication factory models in which parental DNA enters a replication complex containing associated replisomes on sister forks and from which newly replicated DNA exits prior to segregation.

The mean time between replisome appearance at *oriC* and disappearance in the *ter* region is 65 min, which will be close to S phase since replication initiation occurs within 5 min of replisome assembly and disassembly of replisome foci occurs just a few min before *ter3* sister focus separation. Cell division occurs ∼30 min after replisome disassembly; this G2 period allows completion of unlinking and segregation of sister chromosomes and cell division. Our estimates for cohesion at *ori1*, L3-R3, and *ter3* fall in the range 3 min–16.5 min, as judged by the time of *ori1* sister locus separation as compared to the time of replisome appearance-sister focus separation, the estimated time of L3-R3 replication, or the time of replisome disappearance with respect to *ter3* duplication. These values are in the range observed for several other studies, which have led to the proposals that genetic loci segregate sequentially and progressively as replication proceeds, with any cohesion limited to small fractions of S phase and the cell cycle (Fekete and Chattoraj, 2005; Nielsen et al., 2006a; Viollier et al., 2004; Wang et al., 2005), This view requires no dedicated cohesion machinery and is most parsimoniously explained if the independent progress of each replisome along DNA allows some rotation at the fork as it proceeds, thereby generating precatenanes that would interlink newly replicated sisters, until decatenation by toposoisomerases. In contrast, some other studies have proposed that, as in eukaryotes, there is extensive cohesion between newly replicated sisters over much of their length and for much of the *E. coli* cell cycle, with consequently no necessary mechanistic and temporal relationship between replication and segregation (Bates and Kleckner, 2005; Sunako et al., 2001).

### Cellular Marks, Replication Factories, and the Replisome

The coherent view of replication organization and replisome action presented here is very different from that which has emerged from many other studies that have been interpreted within the framework of the replication factory model (Dingman, 1974). This model evolved from the proposal that membrane-associated origins and replication forks could organize replication of the *E. coli* chromosome and facilitate its segregation (Jacob et al., 1963). In the model, the two replisomes diverging from a given origin remain together, with parental DNA moving into the fixed factory to replicate and sister chromosomes leaving after replication. It has been proposed that fixed replication factories may help organize the replication machinery and substrates and avoid DNA entanglement (Cook, 1999; Hearst et al., 1998; Hozak et al., 1993). Strong evidence for factories in yeast comes from timelapse studies that have shown that the two forks diverging from a given origin remain associated with each other and with loci equidistant from the origin; some ten such replisome pairs constitute a single replication factory (Kitamura et al., 2006).

Our results are contrary to both central assumptions of the factory model: that the two sister forks derived from a given replication initiation remain close together and that they occupy a fixed cell position into which parental DNA enters and newly replicated DNA exits. In conditions where only two sister replication forks are present, we observe two dynamic replisome foci behaving independently in separate cell halves for almost all of S phase with an overall progression outwards as replication proceeds, followed by an inwards movement as termination approaches. This observation is consistent with studies showing the functional independence of sister forks (Breier et al., 2005; Possoz et al., 2006).

Since sister forks are spatially separate for most of S phase and are not obviously fixed to any structure of the cell other than DNA, we propose that the only determinant of the replication position is DNA and that the replisomes track along DNA as replication proceeds. The dynamic movement of the replisome, which exhibits a ∼7–10-increased diffusion coefficient in the cell long axis, as compared to L3 or R3 loci that are not undergoing replication segregation, strongly supports this view. Furthermore, the observed rates of replisome movement (mean step sizes of ∼100 nm/3 s and a D_app_ of ∼(5–7 × 10^2^) nm^2^/s) are of the order one would predict for the replication of ∼450 × 10 kb independent chromosomal domains since such domains would have a diameter of ∼100 nm and take ∼12 s to replicate. Assuming L3-R3 movement reflects in large part domain movement, we expect chromosome domain mobility on average to be 7- to 10-fold less than that of the replisome. We note that loci undergoing replication may exhibit different dynamics, although we would expect such dynamics to be constrained by surrounding nonreplicating domains; analysis of this is not yet technically feasible.

We observed that sister forks sometimes come sufficiently close together to be observed as one focus close to midcell, before separating back toward the quarter positions. A similar oscillation has been observed in *B. subtilis*, although the foci, when separate, remain close together in the midcell region (Berkmen and Grossman, 2006; Migocki et al., 2004). This splitting and fusion of foci might arise by ongoing reorganization of the growing sister nucleoids and the contracting parental nucleoid as replication proceeds. At termination the two forks come together in the midcell region to form a single focus. Markers that are replicated in the latter part of the replication cycle are located toward a pole prior to replication, with loci replicated by separate forks being at opposite poles and therefore outside of the average replisome position (Figure 6). Ongoing reorganization of DNA as replication proceeds may explain why replisomes are rarely at the outer nucleoid edges.

Our demonstration that replisome components assemble at *oriC* irrespective of its position in the cell also argues against the factory model. A corollary of this observation is that replication can, in principle, initiate at any origin in the cell, irrespective of its position, whether it be plasmid, viral, or chromosomal. We have no reason to believe that anything other than a functional origin and active initiator proteins determine the timing and position of replisome assembly and concomitant initiation. Work with *B. subtilis* led to a similar conclusion (Berkmen and Grossman, 2006).

In contrast to the conclusions here, many earlier reports suggested that bacterial replisomes assemble and position themselves according to unknown spatial determinants in the cell, and that the replication origins move to these positions at the time of initiation (Bates and Kleckner, 2005; den Blaauwen et al., 2006; Lau et al., 2003; Migocki et al., 2004). Furthermore, many reports of localization of active replication have been interpreted in terms of stationary replication factories (Lemon and Grossman, 1998, 2000; Meile et al., 2006; Molina and Skarstad, 2004; Sawitzke and Austin, 2001), although transient separation of replisome reporters in the midcell region has been reported (Berkmen and Grossman, 2006; Migocki et al., 2004), and the idea of “translocating replication factories” has been proposed (Hiraga et al., 2000; Jensen et al., 2001; Kongsuwan et al., 2002).

In a thorough immunocytochemical and FISH study of replication and chromosome organization in fixed *E. coli* derived from synchronous cultures grown at 30°C, Bates and Kleckner (2005) observed the splitting of a single DnaX replisome focus at midcell into two occupying separate cell halves, although this occurred ∼40% through S phase, ∼10 min after *ori* separation, rather than before *ori* separation in early S phase as measured directly here. This work also reported the DNA- and *oriC*-independent assembly of DnaX foci at midcell long before replication initiation, with *oriC* apparently moving to the replisome at initiation. We can provide no explanation for the differences in conclusions from this study of fixed cells and those reported here. A strength of our study is the use of timelapse in live cells that allows us to follow individual cells from birth to division and from replication initiation to termination, thus circumventing the need to synchronize cells. Because the work of Bates and Kleckner used fixed cells, it was important for them to use cells from “unperturbed” synchronous cultures obtained by eluting new-born cells from a column. Furthermore, in our work, the same live cells and direct microscopic assays are used to provide simultaneous insight into replisome behavior, S-G2 phases of the cell cycle, and sister cohesion of the *ori1*, L3, R3, and *ter3* genetic loci. It is noteworthy that our demonstration that replisome components assemble at *oriC* at initiation is based on experiments with wild-type and Muk^−^ cells as well as *dnaC*^ts^ cells synchronized for replication by temperature shift. Independent replisome action is not dependent on growth rate or temperature (R.R-L. and D.J.S., unpublished data) and is, we believe, unlikely to be strain dependent.

In most previous work, different assays have been used to assess replication time and locus cohesion, and often replisome positioning and number have not been compared with genetic loci or other parameters. Therefore, the interpretations of this work have often been clouded by uncertainties about the number of active forks and other cell-cycle parameters. Moreover, FISH and immunocytochemical techniques can underestimate the number of genetic loci or replisomes, while the binding of fluorescent proteins to DNA, used to visualize genetic loci, may perturb replication or segregation of a locus (Possoz et al., 2006; Woldringh and Nanninga, 2006). The use of fixed cells for FISH or immunocytochemistry gives no direct information on dynamics and in our experience is more subject to artifact than live-cell imaging. Both timelapse and snapshot analysis were used here in order to compensate for limitations in either technique. Even in live-cell imaging, the use of timelapse is crucial for gaining insight into the dynamics of the system and for determining the sequence of cell-cycle-dependent events.

### Independent Forks and Nucleoid Replichore Organization

Our earlier work, using simultaneous tracking of two genetic markers, showed that the nucleoid in living cells is organized with *oriC* close to midcell, and the separate left and right chromosome arms disposed on each side of the *oriC* and the terminus region stretching from the left edge of the nucleoid to the right edge. Unexpectedly, replication segregation created a < left-*ori*-right-left-*ori*-right > organization in the majority of sister nucleoids (Wang et al., 2006). To explain this organization, a “replisome splitting” model was proposed in which the pattern of segregation of sister chromosomes is achieved by the replication forks separating into two different cell halves after initiation (Wang et al., 2006). The results here provide a strong experimental basis for supporting this model and for discarding the idea that *E. coli* replication occurs in fixed replication factories. The replication-dependent movement apart of the sister replisomes into separate cell halves after initiation is likely the direct consequence of the replisomes tracking along the left and right replichores and may help direct the subsequent bidirectional segregation of newly replicated DNA.

## Experimental Procedures

### Bacterial Strains, Growth, and Microscopy

*E. coli* K12 AB1157 strains had chromosomal loci marked with *tetO* and *lacO* arrays (Lau et al., 2003; Wang et al., 2005, 2006). To study the localization of *ori* and *ter*, arrays of 240 *tetO* sites,15 kb counterclockwise from *oriC* (*ori1*) or 50 kb clockwise from *dif* (*ter3*), were used. Other arrays were at L3 (2268 kb; 240 *tetO*) and R3 (852 kb; 240 *lacO*) (Wang et al., 2006). Expression of TetR-mCerulean and LacI-mCherry (Rizzo et al., 2004; Shaner et al., 2004) was by inserting fusion genes, regulated by the *lac* promoter, into *galK* or *leuB* (Supplemental Experimental Procedures). Fluorescent replisome fusions were to the C terminus of the endogenous gene with a 14 aa linker. A strain containing an ectopic copy of *ssb-cfp* was a gift of A. Wright. The very bright YPet fluorescent protein was used to visualize replisome components when possible (Nguyen and Daugherty, 2005). The *ssb-ypet* strain showed wild-type cell-cycle parameters but some genetic instability. Expression from pLAU51 was used to stall replication forks (Lau et al., 2003). Cells were grown at 37°C in LB or in M9 supplemented with 0.2% of glycerol and essential nutrients (Lau et al., 2003). For microscopy, cells were grown at 37°C and subcultured once in M9-Gly without antibiotics. One hundred ng/ml of AT was added when binding of TetR-CFP to *tetO* arrays was used to visualize genetic loci. For microscopy, cells were grown to A_600_ 0.1–0.2 and laid onto an M9-Gly 1% agarose pad on a slide. Cells were visualized with a 100× objective on a Nikon Eclipse TE2000-U microscope, equipped with a Photometrics Cool-SNAP HQ CCD camera. Images were analyzed and processed by Metamorph 6.2.

## Supplemental Data

Document S1. Supplemental Experimental Procedures, Four Figures, and One Reference

Movie S1 Inhibition of DNA replication by hydroxyurea (HU; 100 mM) inhibits replisome splitting (green) after initiation and *ori1* segregation (red). Forty-five minute timelapse (5 min intervals) of a cell that has assembled a replisome (Ssb) at *oriC*. Note that replisome and *ori1* colocalize throughout the movie and that their motion is correlated.

## References

[bib1] Adachi S., Kohiyama M., Onogi T., Hiraga S. (2005). Localization of replication forks in wild-type and *mukB* mutant cells of *Escherichia* coli. Mol. Genet. Genomics.

[bib2] Bates D., Kleckner N. (2005). Chromosome and replisome dynamics in *E. coli*: loss of sister cohesion triggers global chromosome movement and mediates chromosome segregation. Cell.

[bib3] Berkmen M.B., Grossman A.D. (2006). Spatial and temporal organization of the *Bacillus subtilis* replication cycle. Mol. Microbiol..

[bib4] Breier A.M., Weier H.-U.G., Cozzarelli N.R. (2005). Independence of replisomes in *Escherichia coli* chromosomal replication. Proc. Natl. Acad. Sci. USA.

[bib5] Cook P.R. (1999). The organization of replication and transcription. Science.

[bib6] Danilova O., Reyes-Lamothe R., Pinskaya M., Sherratt D., Possoz C. (2007). MukB colocalizes with the *oriC* region and is required for organization of the two *Escherichia coli* chromosome arms into separate cell halves. Mol. Microbiol..

[bib7] den Blaauwen T., Aarsman M.E.G., Wheeler L.J., Nanninga N. (2006). Pre-replication assembly of *E. coli* replisome components. Mol. Microbiol..

[bib8] Deng S., Stein R.A., Higgins N.P. (2005). Organization of supercoil domains and their reorganization by transcription. Mol. Microbiol..

[bib9] Dingman C.W. (1974). Bidirectional chromosome replication: Some topological considerations. J. Theor. Biol..

[bib10] Elmore S., Muller M., Vischer N., Odijk T., Woldringh C.L. (2005). Single-particle tracking of oriC-GFP fluorescent spots during chromosome segregation in *Escherichia coli*. J. Struct. Biol..

[bib11] Fekete R., Chattoraj D. (2005). A cis-acting sequence involved in chromosome segregation in *Escherichia coli*. Mol. Microbiol..

[bib12] Fiebig A., Keren K., Theriot J.A. (2006). Fine-scale time-lapse analysis of the biphasic, dynamic behaviour of the two *Vibrio cholerae* chromosomes. Mol. Microbiol..

[bib13] Hearst J.E., Kauffman L., McClain W.M. (1998). A simple mechanism for the avoidance of entanglement during chromosome replication. Trends Genet..

[bib14] Hiraga S., Ichinose C., Onogi T., Niki H., Yamazoe M. (2000). Bidirectional migration of SeqA-bound hemimethylated DNA clusters and pairing of oriC copies in *Escherichia coli*. Genes Cells.

[bib15] Hozak P., Hassan A.B., Jackson D.A., Cook P.R. (1993). Visualization of replication factories attached to nucleoskeleton. Cell.

[bib16] Jacob F., Brenner S., Cuzin F. (1963). On the regulation of DNA replication in bacteria. Cold Spring Harb. Symp. Quant. Biol..

[bib17] Jensen R.B., Wang S.C., Shapiro L. (2001). A moving DNA replication factory in *Caulobacter crescentus*. EMBO J..

[bib18] Kitamura E., Blow J.J., Tanaka T.U. (2006). Live-cell imaging reveals replication of individual replicons in eukaryotic replication factories. Cell.

[bib19] Kongsuwan K., Dalrymple B.P., Wijffels G., Jennings P.A. (2002). Cellular localisation of the clamp protein during DNA replication. FEMS Microbiol. Lett..

[bib20] Lau I.F., Filipe S.R., Soballe B., Okstad O.A., Barre F.X., Sherratt D.J. (2003). Spatial and temporal organization of replicating *Escherichia coli* chromosomes. Mol. Microbiol..

[bib21] Lemon K.P., Grossman A.D. (1998). Localization of bacterial DNA polymerase: evidence for a factory model of replication. Science.

[bib22] Lemon K.P., Grossman A.D. (2000). Movement of replicating DNA through a stationary replisome. Mol. Cell.

[bib23] Maisnier-Patin S., Nordstrom K., Dasgupta S. (2001). Replication arrests during a single round of replication of the *Escherichia coli* chromosome in the absence of DnaC activity. Mol. Microbiol..

[bib24] Marshall W.F., Straight A., Marko J.F., Swedlow J., Dernburg A., Belmont A., Murray A.W., Agard D.A., Sedat J.W. (1997). Interphase chromosomes undergo constrained diffusional motion in living cells. Curr. Biol..

[bib25] Meile J.C., Wu L.J., Ehrlich S.D., Errington J., Noirot P. (2006). Systematic localisation of proteins fused to the green fluorescent protein in *Bacillus subtilis*: identification of new proteins at the DNA replication factory. Proteomics.

[bib26] Migocki M.D., Lewis P.J., Wake R.G., Harry E.J. (2004). The midcell replication factory in *Bacillus subtilis* is highly mobile: implications for coordinating chromosome replication with other cell cycle events. Mol. Microbiol..

[bib27] Molina F., Skarstad K. (2004). Replication fork and SeqA focus distributions in *Escherichia coli* suggest a replication hyperstructure dependent on nucleotide metabolism. Mol. Microbiol..

[bib28] Nguyen A.W., Daugherty P.S. (2005). Evolutionary optimization of fluorescent proteins for intracellular FRET. Nat. Biotechnol..

[bib29] Nielsen H.J., Li Y., Youngren B., Hansen F.G., Austin S. (2006). Progressive segregation of the *Escherichia coli* chromosome. Mol. Microbiol..

[bib30] Nielsen H.J., Ottesen J.R., Youngren B., Austin S.J., Hansen F.G. (2006). The *Escherichia coli* chromosome is organized with the left and right chromosome arms in separate cell halves. Mol. Microbiol..

[bib31] O'Donnell M. (2006). Replisome architecture and dynamics in *Escherichia coli*. J. Biol. Chem..

[bib32] Possoz C., Filipe S.R., Grainge I., Sherratt D.J. (2006). Tracking of controlled *Escherichia coli* replication fork stalling and restart at repressor-bound DNA in vivo. EMBO J..

[bib33] Postow L., Hardy C.D., Arsuaga J., Cozzarelli N.R. (2004). Topological domain structure of the *Escherichia coli* chromosome. Genes Dev..

[bib34] Rizzo M.A., Springer G.H., Granada B., Piston D.W. (2004). An improved cyan fluorescent protein variant useful for FRET. Nat. Biotechnol..

[bib35] Sawitzke J., Austin S. (2001). An analysis of the factory model for chromosome replication and segregation in bacteria. Mol. Microbiol..

[bib36] Shaner N.C., Campbell R.E., Steinbach P.A., Giepmans B.N., Palmer A.E., Tsien R.Y. (2004). Improved monomeric red, orange and yellow fluorescent proteins derived from Discosoma sp. red fluorescent protein. Nat. Biotechnol..

[bib37] Sunako Y., Onogi T., Hiraga S. (2001). Sister chromosome cohesion of *Escherichia coli*. Mol. Microbiol..

[bib38] Viollier P.H., Thanbichler M., McGrath P.T., West L., Meewan M., McAdams H.H., Shapiro L. (2004). Rapid and sequential movement of individual chromosomal loci to specific subcellular locations during bacterial DNA replication. Proc. Natl. Acad. Sci. USA.

[bib39] Wang X., Possoz C., Sherratt D.J. (2005). Dancing around the divisome: asymmetric chromosme segregation in *Escherichia coli*. Genes Dev..

[bib40] Wang X., Liu X., Possoz C., Sherratt D.J. (2006). The two *Escherichia coli* chromosome arms locate to separate cell halves. Genes Dev..

[bib41] Withers H.L., Bernander R. (1998). Characterization of *dnaC2* and *dnaC28* mutants by flow cytometry. J. Bacteriol..

[bib42] Woldringh C.L., Nanninga N. (2006). Structural and physical aspects of bacterial chromosome segregation. J. Struct. Biol..

[bib43] Zimmerman S.B. (2006). Shape and compaction of *Escherichia coli* nucleoids. J. Struct. Biol..

