# Supplementary material for: Independent Positioning and Action of Escherichia coli Replisomes in Live Cells
Source: Cell. 2008 Apr 4;133(1):90–102. doi: 10.1016/j.cell.2008.01.044 (PMC2288635; doi:10.1016/j.cell.2008.01.044)
Supplement: Document S1. Supplemental Experimental Procedures, Four Figures, and One Reference [file mmc1.pdf]

Cell, Volume *133*

Supplemental Data

Independent Positioning and Action of *Escherichia coli* Replisomes in Live Cells

Rodrigo Reyes-Lamothe, Christophe Possoz, Olessia Danilova, and David J. Sherratt

Supplemental Experimental Procedures

Construction of chromosomal ypet fusions

The gene encoding the fluorescent protein YPet was amplified by PCR. The forward primer was preceded by a sequence coding for an 11 amino acid linker and a SacI site (5'-aaggagctcggctggctccgctgctggttctggcgaattcatgtctaaaggtgaagaatta-3'). The reverse primer included an XmaI restriction site (5'-taggctcccgggttatttgtacaattcattcatacc-3'). A kanamycin resistance cassette, flanked by *frt* sites, was amplified from pKD4 using the primers described elsewhere (Datsenko and Wanner, 2000). The fragments were cloned in pUC18 between SacI and BamHI sites resulting in plasmid pROD10.

Oligonucleotides used had a 50 nt complementary sequence to the last 50 bp of the gene (not including the stop codon) or to the sequence immediately downstream of it, followed by 20 nt complementary to the linker sequence 5' of *ypet* or the end of the kanamycin resistance cassette in pROD10 respectively. Primer sequences used in the construction of fusion strains are:
ssb-ypet (forward: 5'-cagcgccgtctaacgagccgccgatggactttgatgatgacattccgttctcggctggctccgctgc–3';
reverse: 5'-ctttaatcatccaccttaaaacaatataacctattgttttaatgacaaatcttatgaatatcctccttag–3');
dnaQ-ypet (forward: 5'-cccgtctcgatctggtgcagaagaaaggcggaagttgcctctggcgagcatcggctggctccgctgc –3';
reverse: 5'-tgctcgaaaaatcgcccaagtcgctatttttagcgcctttcacaggtattcttatgaatatcctccttag–3'); and
holC-ypet (forward: 5'-ataaagcctaccgcgtggctggtttcaacctgaatacggcaacctggaaatcggctggctccgctgc–3';
reverse: 5'-tgctcgtaaagcggctgttcgatatcttgtgggttatatgtcttttccatcttatgaatatcctccttag–3').

The polymerase chain reaction (PCR) used template DNA pROD10. The DNA fragment was gel purified and ~1 μg was used for electroporation of AB1157 cells overexpressing λ-Red proteins from pKD46 (Datsenko and Wanner 2000). The correct insertion of the fragment into the chromosome was assayed by PCR.

Construction of chromosomal encoded fluorescent repressors

Copies of the chimeric genes *tetR-mcerulean* and *lacI-mcherry* were integrated into the chromosome in order to avoid the use of plasmids, which could potentially alter the localization of the replisome.

For *tetR-mcerulean*, the gene *mcerulean* was amplified using the primers:
5'-aaggagctcggctggctccgctgctgtgagcaagggcgaggag-3' (forward) and
5'-taggctcccgggttacttgtacagctcgtcc-3'(reverse), resulting in a fragment containing the gene preceded by the sequence of a 6aa flexible linker. It was then cloned in pUC18 between SacI and BamHI sites along with a kanamycin resistance cassette obtained using

the primers described above. The resulting plasmid was pROD7. The gene *tetR* was then amplified using the forward and reverse primers 5'-cttgaattcgtctagattagataaaagtaa-3'and 5'-cttgagctcgaaatgtcagacccactttcacatttaa-3' respectively, and cloned in pROD7 between EcoRI and SacI sites resulting in pROD14, which has an in-frame fusion regulated by a *lac* promoter. The promoter, gene fusion and resistance cassette were integrated in the chromosome by replacing the gene *galK* through λ-Red recombination, using the primers 5'-gtttgcgcgcagtcagcgatatccattttcgcgaatccggagtgtaagaacgcccaatacgcaaaccg-3'and 5'-cggctgaccatcgggtgccagtgcgggagtttcgttcagcactgtcctgccttatgaatatcctccttag-3'.

In the case of *lacI-mcherry*, a similar strategy to that of *tetR-mcerulean* was used, using the same primers to amplify the fluorescent protein, and the gene was cloned into pUC18 along with a resistance cassette for chloramphenicol (template pKD3, using the same primers as for kanamycin resistance), this plasmid was named pROD22. *lacI* was amplified using the primers: 5'-cttgaattcggtgaatgtgaaaccagtaacgttat-3'and 5'-ttagagctcgaacccagctgcattaatgaatc-3', and cloned in pROD22 between EcoRI and SacI sites, resulting in pROD25. The fusion was inserted in the chromosome by replacing *leuB* using the primers:
5'-aaagagttgcaacgcaaagctcaacacaacgaaaacaacaaggaaaccgtcgcccaatacgcaaaccg-3' and 5'-gtcgaacaatttttcgtataacgtcttagccatgattacacccccttctgccttatgaatatcctccttag -3'.

Microscopy

For snapshot analysis, cultures were grown overnight in M9 with glycerol as carbon source and subcultured once in the same medium. Cells were obtained from cultures in the early logarithmic phase (A_{600} 0.1 and 0.2), concentrated, resuspended in PBS and laid on a 1 % agarose pad on a slide. Nucleoids were visualized by staining them with 4',6-Diamidino-2-phenylindole (DAPI) (final concentration of 1 μg/ml). For timelapse, cells were treated as before but were instead laid on an M9-glycerol 1% agarose pad. Slides were incubated for 30 minutes in the microscope incubator chamber at the temperature used in the respective experiment. Cells were visualized with a 100x objective on a Nikon Eclipse TE2000-U microscope, equipped with a Photometrics Cool-SNAP HQ CCD camera. Images were taken, analyzed and processed by Metamorph 6.2.

Flow cytometry

Cells were fixed with 70% v/v ethanol. Samples were washed twice with PBS and stained with Syto-16 (0.1 μg/ml). 100 000 events recorded in a Becton Dickinson FACScalibur machine using FL1-H.

Supplemental References

Datsenko, K.A., and Wanner, B.L. (2000). One-step inactivation of chromosomal genes in *Escherichia coli* K-12 using PCR products. Proc. Natl. Acad. Sci. U S A *97*, 6640-6645.

Figure S1. Growth and cell cycle parameters for replisome-fluorescent protein fusions

Fusions to Ssb, DnaQ, HolC, DnaX, HolA, HolD and DnaE not only had generation times close to the wild type strain AB1157, but showed wild type flow cytometry profiles. Furthermore, in microscopy snapshots of steady state cells, the cell length profiles and relative proportions of 1 *ori1* to *2 ori* foci cells (when *ori1* was labeled) was in the same range as for wild-type. This relative proportion gives a good indication of cell cycle parameters. For example, in Fig. 3D, 29% of *ori1* DnaQ-YPet cells had a single *ori1* focus as compared to 23% in Wang et al. (2005), while 24.7% of Ssb-YPet cells had 1 *ori1* and 28.5% of HolC-YPet cells had 1 *ori1* focus (not shown). Although viable, fluorescent DnaB fusions were not analyzed extensively because they grew poorly sometimes and then had less normal cell cycle parameters. Fusions to DnaG and DnaN were not viable.

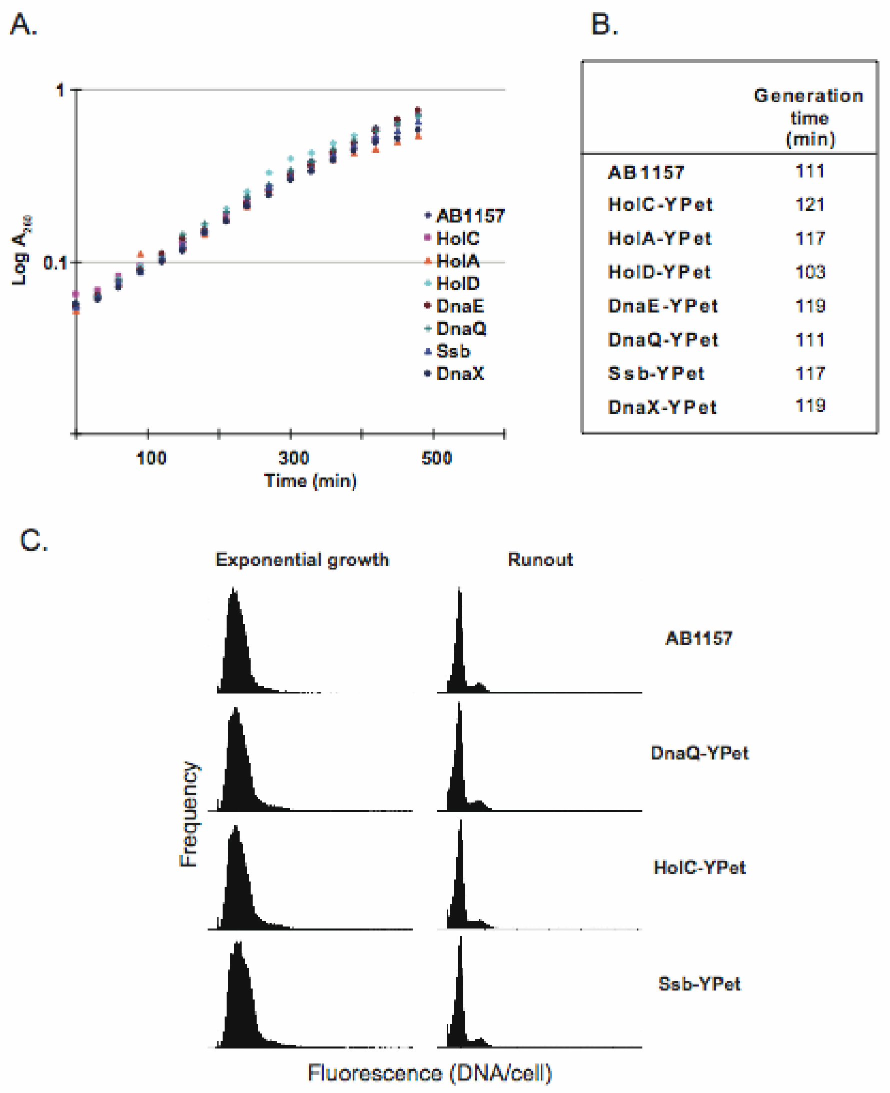

Figure S1. Reyes-Lamothe *et al.*

Figure S2.

A. DnaQ and Ssb focus frequency in *dnaA*^{ts} and *dnaC*^{ts} strains, with corresponding flow cytometry analysis after runout at 42° C (see Fig. 2).

B. Correlation between *ori1* and Ssb positioning at the time of Ssb focus appearance. See Fig. 2B.

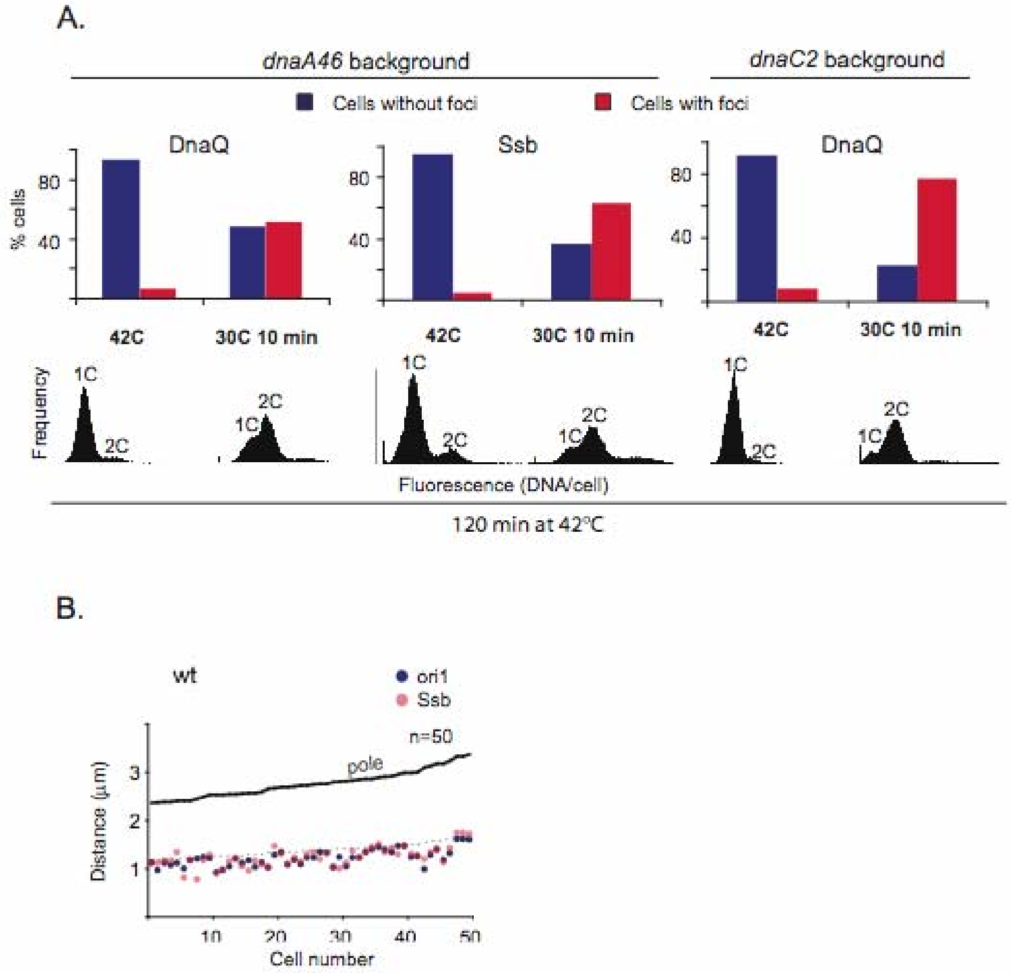

Figure S2. Reyes-Lamothe *et al.*

Figure S3. Ssb trajectories for 12/50 timelapse series
See Fig. 3. Examination shows that sister replisomes are each mobile with respect to midcell. An analysis of sister replisome mobility is given in Fig. 6 and associated text.

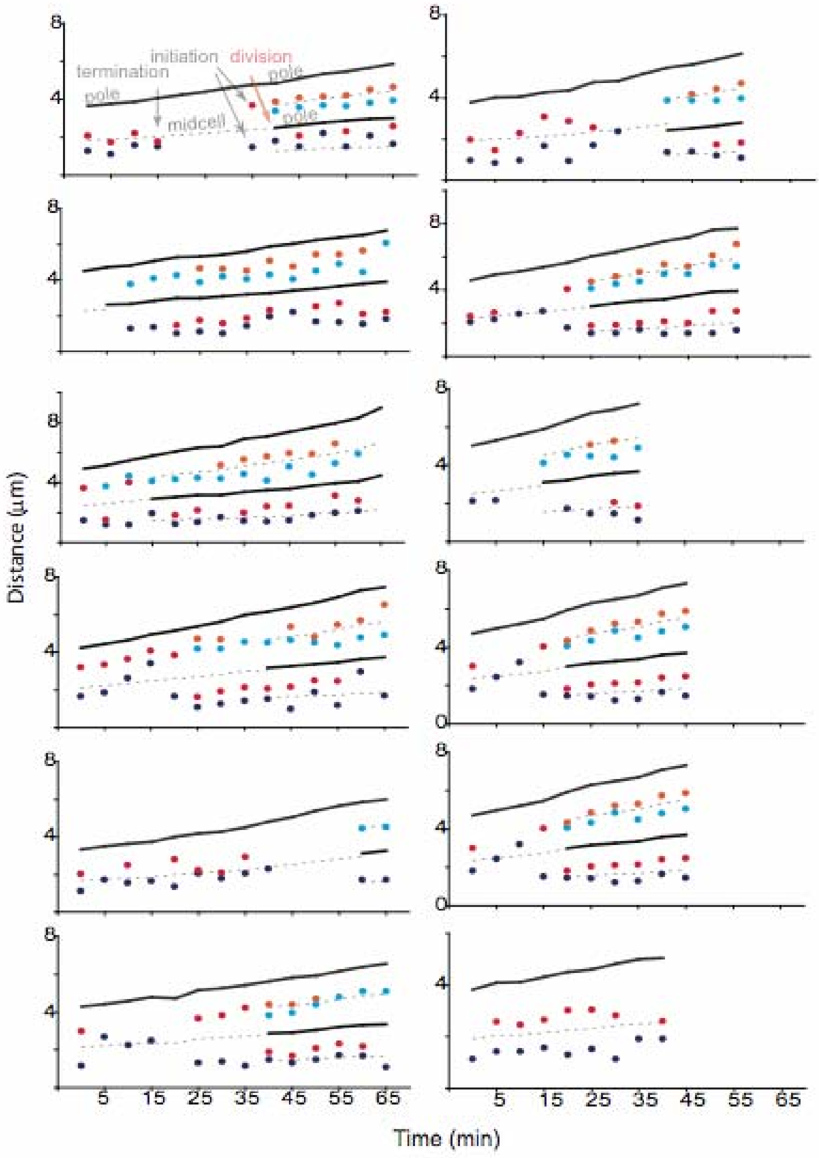

Figure S3. Reyes-Lamothe *et al.*

Figure S4. Ssb-L3-R3 trajectories

A map of the loci used is shown (top). See Figure 6A. Examination of sister foci with respect to midcell provides insight into the relative mobility of sister replisomes.

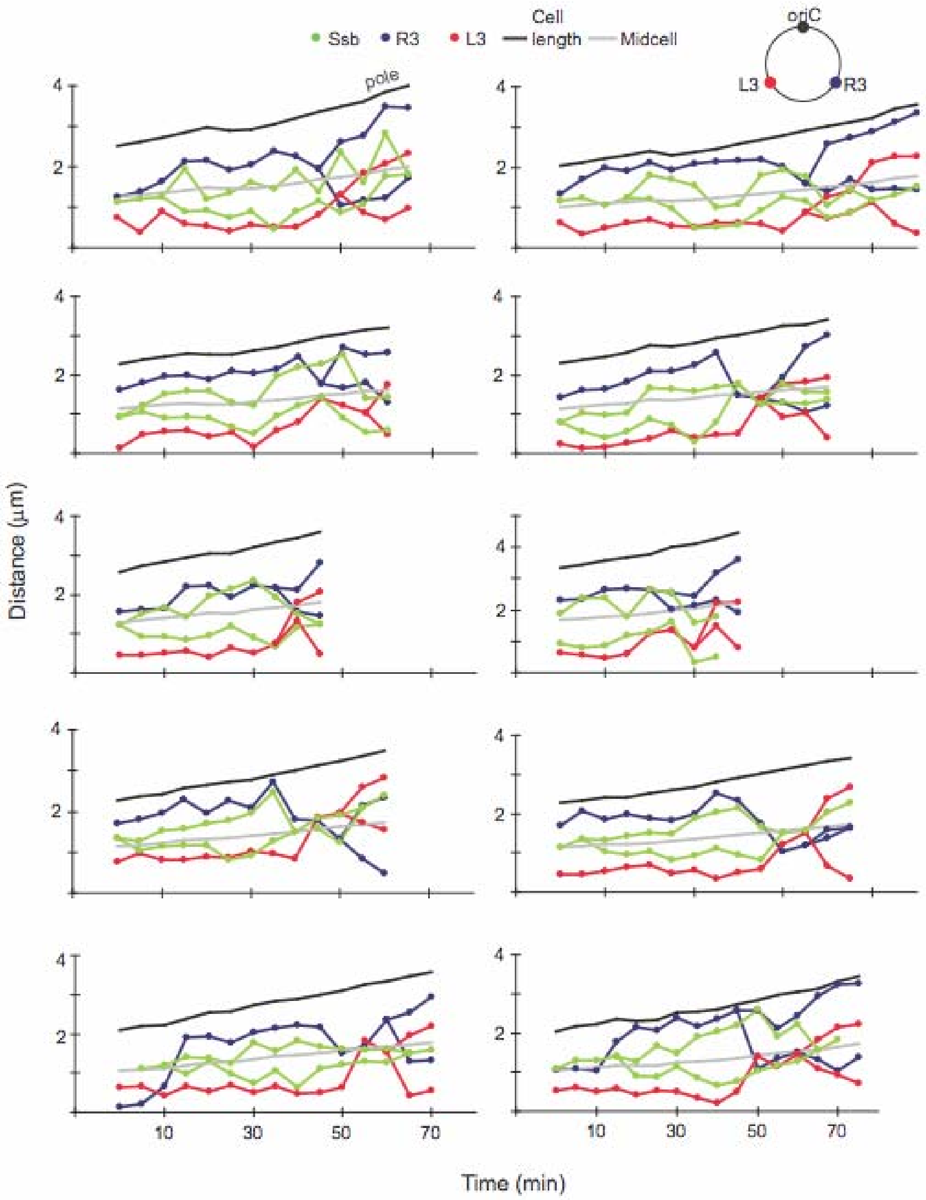

Figure S4. Reyes-Lamothe *et al.*
